# Exopolysaccharide produced from Lactiplantibacillus plantarum HAN99 and its nanoparticle formulations in agricultural applications

**DOI:** 10.1038/s41598-025-03913-9

**Published:** 2025-06-01

**Authors:** Hania M. El-Messiry, Amira M. Hamdan, Nevine B. Ghanem, Mohamed Hagar

**Affiliations:** 1 Botany and Microbiology Department, Faculty of Science, Alexandria University, Alexandria, Egypt; 2 Oceanography Department, Faculty of Science, Alexandria University, Alexandria, Egypt; 3 Chemistry Department, Faculty of Science, Alexandria University, Alexandria, Egypt

**Keywords:** *Lactiplantibacillus plantarum* HAN99, Marine lactic acid bacteria, Polysaccharides Chitosan-based nanoparticles, Plant growth promoting, Sustainable agricultural methods, Microbiology, Applied microbiology, Environmental microbiology

## Abstract

In this study, *Lactiplantibacillus* plantarum HAN99, isolated from sediment samples collected along the Alexandria Mediterranean Seacoast in Egypt, was evaluated for its ability to produce polysaccharides. To optimize polysaccharide production, statistical techniques were used, and the extracted polysaccharides were purified for further characterization. High-Performance Liquid Chromatography (HPLC) analysis identified glucose and galactose as the primary components of the polysaccharide. These polysaccharides were then loaded onto chitosan-based nanoparticles, which were characterized using Fourier Transform-Infrared Spectroscopy (FT-IR) and scanning electron microscopy (SEM). The study further investigated the potential agricultural applications of the polysaccharide-loaded nanoparticles by assessing their effects on plant growth. The results revealed that the nanoparticles enhanced the growth of Mentha (mint) leaves, reducing leaf loss compared to the control group. Additionally, the EPS chitosan-based nanoparticles exhibited strong antioxidant activity, as demonstrated by a DPPH assay (∼75.6–80.3%). These findings highlight the potential of microbial polysaccharides as sustainable, eco-friendly alternatives for agricultural enhancement and the development of green agricultural practices.

**Supplementary Information:**

The online version contains supplementary material available at 10.1038/s41598-025-03913-9.

## Introduction

Agriculture sustainability has become a necessity in recent years, due to the rapid development of the human population, the abiotic and biotic stress factors on plants resulting from climate change^1^, and the scarcity of fertile lands^2^. Climate change, in particular, has a significant effect on the field of agriculture due to fluctuations in the amount of annual rainfall, variations in the average temperature, severe heat waves, and alterations in the concentration of atmospheric CO_2_^3,4^. These impacts represent a significant risk to food availability in the upcoming years, and researchers have been concerned about them for decades, particularly in developing countries where malnutrition remains a persistent issue.

To tackle these challenges and deal with the rapid increase in the world population, solutions to increase the production of crops and ensure there are sufficient quantities of food are being rapidly studied and considered. While using agrochemicals has been proven to increase crop production and reduce plant losses to diseases^5^, it has also been associated with increased risks to the surrounding environment and ecosystems^6^, along with water and soil pollution^7,8^. Furthermore, agrochemicals are considered an expensive solution for boosting crop growth. There is a growing emphasis on investigating alternative approaches to enhance crop growth while ensuring sustainability.

Biopolymers, particularly polysaccharides, have emerged as sustainable alternatives to synthetic agrochemicals in modern agriculture. Their biodegradable nature and ability to enhance soil structure, water retention, and nutrient availability make them valuable for improving crop productivity and soil health. Recent studies have highlighted the potential of biopolymers to serve as biofertilizers and biostimulants, promoting plant growth and resilience to environmental stresses^9,10^.

Lactic acid bacteria attracted the attention of researchers due to their ability to produce a wide range of polysaccharides^11^. These Gram-positive anaerobic bacteria utilize carbohydrates as their principal carbon source, leading to the production of various metabolites, including vitamins, bacteriocins, and polysaccharides^12^.

Researchers commonly use the lactic acid genera *Lactococcus*, *Streptococcus*, *Lactobacillus*, and *Pediococcus* to produce polysaccharides. Lactic acid bacteria are widely distributed in nature and can be isolated from diverse sources, including marine environments^13^. Marine strains produce microbial polysaccharides, a promising alternative to traditional agrochemicals, and can be used to control bacterial phytopathogens^14^. They can also be used to encapsulate beneficial microorganisms to extend their lifespan till they are applied on infected plants^15^ These techniques have been shown effective when an encapsulated* Bacillus subtilis* strain significantly suppressed *Rhizoctonia solani*^16^.

*Lactiplantibacillus plantarum*, in particular, is a bacterium commonly found in fermented foods, plants, and the gastrointestinal tract of humans and animals^17^. It has been recognized for its ability to produce exopolysaccharides (EPS), which possess a range of functional properties^18^. Numerous studies have reported that *L. plantarum* strains synthesize EPS with antioxidant and antimicrobial activities, enhancing their potential for use in agricultural and environmental applications^17^. Such bioactivities suggest promising roles in promoting plant health, supporting beneficial soil microbiota, and contributing to more sustainable farming practices.

Microbial exopolysaccharides can be used as hydrogels in agriculture to improve soil moisture retention and porosity^19^. These 3D hydrophilic networks, obtained from natural polymer materials, can absorb and retain significant quantities of water, boosting crop growth^20^. Superabsorbent polymers were first synthesized in 1938 using divinylbenzene and acrylic acid^21^, and several attempts followed. While they can be based on natural or synthetic monomers, synthetic hydrogels have shown poor degradability, making them a less desirable option.

However, natural hydrogels have demonstrated non-toxic and biodegradable characteristics, making them the most environmentally friendly option^22^. Polysaccharides showed the greatest potential natural polymers that can be used to form hydrogels. Hydrogels can be created by either covalent bonds, non-covalent bonds, or a mix of both, making them a potential option for producing agricultural hydrogels^23^.

In recent years, polysaccharide-based nanoparticles have gained considerable interest as delivery systems in agricultural applications due to their biocompatibility, biodegradability, and tunable physicochemical properties^24^. Their ability to encapsulate active compounds—such as fertilizers, pesticides, or growth promoters—and release them in a controlled manner enables prolonged activity and reduces input frequency. The structural versatility of polysaccharides allows for modifications that enhance encapsulation efficiency, responsiveness to environmental stimuli (e.g., pH or moisture), and target specificity^25^. Furthermore, these nanoformulations are considered environmentally safe, as they degrade into non-toxic byproducts, minimizing ecological risks compared to conventional agrochemicals^22^.

Exploring the agricultural applications of microbial polysaccharides and comprehending their nature will be crucial for altering agricultural practices toward a sustainable and eco-friendly future. This study was designed to assess the efficacy of using microbial polysaccharides derived from marine lactic acid bacteria as hydrogels to improve the growth of crops.

## Results and discussion

### Identification of polysaccharide-producing lactic acid bacteria isolate

Among the purified lactic acid bacterial isolates, HAN99 bacterial culture was selected due to its ability to produce a high content of polysaccharide (61 mg/ml). According to the biochemical, morphological characteristics and 16S rRNA gene sequence analysis, the lactic acid bacterial isolate was identified as *Lactiplantibacillus plantarum* HAN99, and the sequences have been deposited in GenBank under accession numbers PP150039.

### Optimization of polysaccharide (EPS) production by *Lactiplantibacillus plantarum* HAN99

**Fig. 1:**
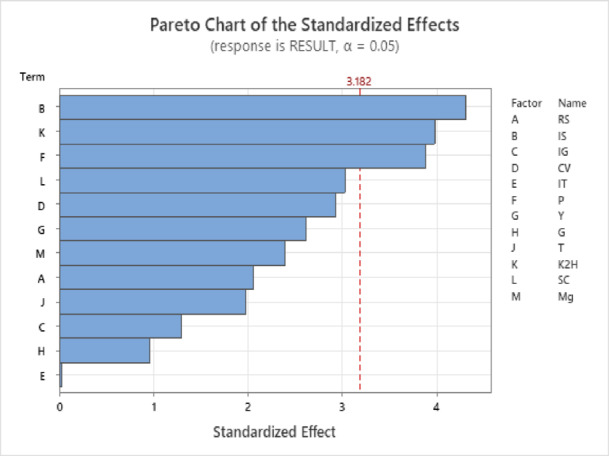
Pareto chart of the significance rank of main effects and interaction effects of different independent variables affecting the production of EPS by *Lactiplantibacillus plantarum* HAN99.

Optimization of nutritional and environmental factors that lead to optimum production of polysaccharides by *Lactiplantibacillus plantarum* HAN99 was carried out using 16 trial runs (Table 1). 12 independent variables were examined in this study: Rate of shaking (RS), Inoculum size (IS), Inoculum age (IG), Culture volume (CV), Incubation time (IT), Peptone (P), Yeast extract (Y), Glucose (G), Tween (T), Dipotassium hydrogen phosphate (K₂H), Sodium acetate (SC), Magnesium sulfate (Mg).).Out of the 12 variables used, three variables showed a significant effect on polysaccharide production, among these, inoculum size was the most significant factor (*P*-value = 0.023). Similar findings were reported by Wang et al.,^26^ where inoculation size was found to be a significant factor in polysaccharide production by *Lactobacillus plantarum* R301. Other significant variables included peptone (*P*-value = 0.030) and K_2_HPO_4_ (*P*-value = 0.028) (Table 2). Peptone was estimated to be an important factor in promoting the production of polysaccharides by *Lactobacillus plantarum* as reported by Wang et al.,^27^. Although incubation temperature was found to have a direct relation to EPS production by lactic acid bacteria multiple times^28,29^, incubation temperature has insignificant effect on polysaccharide production in the present study. The statistical analysis, using the analysis of variance (ANOVA), revealed a robust regression model (R² = 0.96798). The adjusted R-squared value of (0.83392) further highlighted the model’s reliability. Pareto chart shows the sequence of the significant terms and the main interaction effects (Fig. 1), while the normal plot of standardized effects supports the significance of inoculum size, peptone, and K₂HPO₄ (Supplementary Figure. 1).

**Table 1:** Optimization of polysaccharides production by *Lactiplantibacillus plantarum* HAN99 using the Plackett Burman experimental design.

Trial	Factors under study	Independent factors												EPS (mg/ml)
		RS	IS	IG	CV	IT	*P*	Y	G	T	K_2_H	SC	Mg	
1	RS IT G T K_2_H SC	+	-	-	-	+	-	-	+	+	+	+	-	40
2	RS IS P T Mg	+	+	-	-	-	+	-	-	+	-	-	+	50
3	RS IS IG Y SC	+	+	+	-	-	-	+	-	-	-	+	-	70
4	RS IS IG CV G SC Mg	+	+	+	+	-	-	-	+	-	-	+	+	37.6
5	IS IG CV IT T K_2_H	-	+	+	+	+	-	-	-	+	+	-	+	46.6
6	RS IG CV IT K_2_H	+	-	+	+	+	+	-	-	-	+	-	-	110
7	IS CV IT P Y K_2_H SC	-	+	-	+	+	+	+	-	-	+	+	-	10
8	RS IG IT P Y G K_2_H Mg	+	-	+	-	+	+	+	+	-	+	-	+	66.70
9	RS IS CV P Y G T	+	+	-	+	-	+	+	+	+	-	-	-	26.67
10	IS IG IT Y G T K_2_H	-	+	+	-	+	-	+	+	+	+	-	-	40
11	IG CV P G T SC	-	-	+	+	-	+	-	+	+	-	+	-	15
12	RS CV IT Y T K_2_H SC Mg	+	-	-	+	+	-	+	-	+	+	+	+	140
13	IS IT P G K_2_H SC Mg	-	+	-	-	+	+	-	+	-	+	+	+	3.30
14	IG P Y T SC Mg	-	-	+	-	-	+	+	-	+	-	+	+	20
15	CV Y G Mg	-	-	-	+	-	-	+	+	-	-	-	+	190
16	---------------------------	-	-	-	-	-	-	-	-	-	-	-	-	50

**Table 2:** Analysis of variance (ANOVA) for the optimized EPS production by *Lactiplantibacillus plantarum* HAN99.

Source	Coef	Main effect	confidence level	DF	Seq SS	Contribution	Adj SS	Adj MS	F-Value	*P*-Value
Model				12	36740.1	96.80%	36740.1	3061.67	7.56	0.061
Linear				12	36740.1	96.80%	36740.1	3061.67	7.56	0.061
RS	10.38	20.7	86.88655	1	1723.7	4.54%	1723.7	1723.70	4.26	0.131
IS	−21.72	−43.4	97.71004	1	7548.6	19.89%	7548.6	7548.57	18.64	0.023
IG	−6.50	−13.009	71.33357	1	676.9	1.78%	676.9	676.91	1.67	0.287
CV	14.74	29.48	93.89946	1	3477.2	9.16%	3477.2	3477.17	8.58	0.061
IT	−0.17	−0.33	2.437449	1	0.4	0.00%	0.4	0.45	0.00	0.976
P	−19.53	−39.06	96.97202	1	6104.7	16.08%	6104.7	6104.69	15.07	0.030
Y	13.18	26.35	92.09552	1	2779.1	7.32%	2779.1	2779.13	6.86	0.079
G	−4.83	−9.66	59.237	1	373.7	0.98%	373.7	373.75	0.92	0.408
T	−9.96	−19,9	85.78249	1	1586.6	4.18%	1586.6	1586.63	3.92	0.142
K_2_H	20.05	40.09	97.16965	1	6429.2	16.94%	6429.2	6429.23	15.87	0.028
SC	−15.25	−30.5	94.37675	1	3723.1	9.81%	3723.1	3723.14	9.19	0.056
Mg	12.03	24.066	90.33935	1	2316.7	6.10%	2316.7	2316.74	5.72	0.097
Error				3	1215.2	3.20%	1215.2	405.06		
Total				15	37955.3	100.00%				

### Monosaccharide composition analysis of EPS by HPLC

The monosaccharide composition of the polysaccharide was analyzed using High-Performance Liquid Chromatography (HPLC). The analysis of the polysaccharide sample revealed that it consists of glucose and galactose, suggesting it is a heteropolysaccharide, as demonstrated in Supplementary Fig. 2. The assignment of the peaks to glucose and galactose, respectively, was based on the retention times of authenticated reference standards. A mixture of standard monosaccharides, including glucose and galactose, was injected under identical chromatographic conditions, and their retention times were recorded^30^. The retention times of the peaks observed in our sample matched those of the reference standards for glucose and galactose, confirming their identity. Additionally, the peak shapes and relative intensities were consistent with those observed for the monosaccharides in the reference mixture. Similar findings were reported by Wang et al.,^31^ having found glucose and galactose in EPS produced by *Lactobacillus kefiranofaciens* ZW3. Meanwhile, Tallon et al.,^32^ found glucose, galactose, and N-acetylgalactosamine in exopolysaccharides produced by *Lactobacillus plantarum* EP56. Salazar et al.,^33^ found glucose, galactose, and rhamnose in exopolysaccharides by *Lactobacillus* and *Bifidobacterium*, while Marshall et al.,^34^ found rhamnose, glucose, glucosamine, and galactose in the following approximate ratio, 6:5:4:1. Zaghloul and Ibrahim^35^ found rhamnose, galactose, mannose, glucose, and arabinose in exopolysaccharide from *Lactiplantibacillus plantarum* EI6. These findings highlight that the polysaccharide composition can vary among bacterial species, with different strains producing EPS with diverse monosaccharide profiles. However, the polysaccharide sample shares similarities with EPS produced by various *Lactobacillus* species.

While our FT-IR analysis shows the presence of uronic acid, it was not detected by HPLC. This could be explained by the fact that glucose acid derivatives weren’t used as standards for HPLC analysis because of limited availability. As a result, uronic acid, which differs from glucose by the presence of an additional carboxyl group at the sixth carbon, was not effectively detected in the chromatographic profile. The structural differences between glucose and glucuronic acid can lead to variations in retention time and derivatization efficiency, which may cause uronic acid to remain undetected when neutral sugar standards, such as glucose, are used for calibration^36^.

### FT-IR spectrum analysis of the polysaccharide of *Lactiplantibacillus plantarum* HAN99

FT-IR spectrum analysis of the polysaccharide derived from *Lactiplantibacillus plantarum* HAN99 and the chitosan-based nanoparticles was performed to identify the functional groups.

**Fig. 2:**
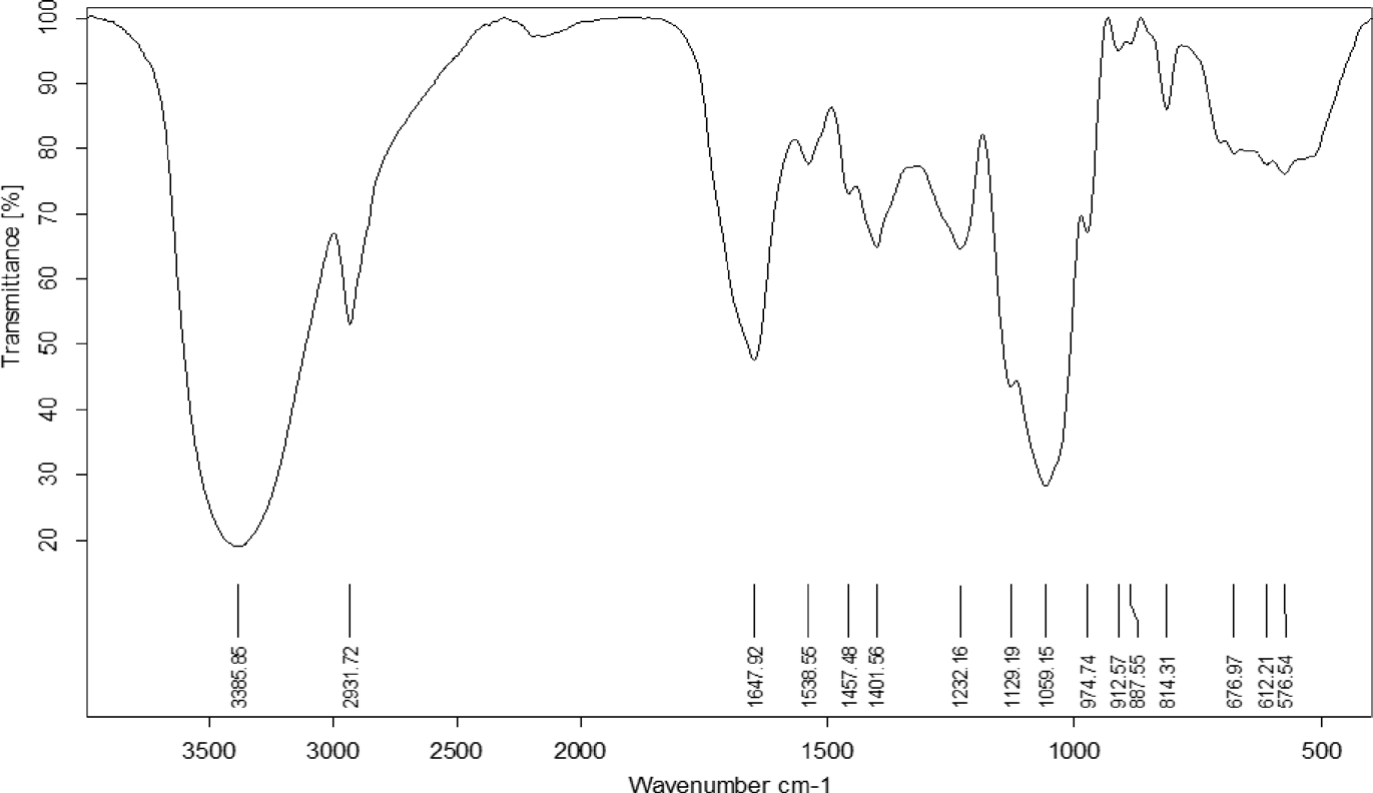
FT-IR analysis of *Lactiplantibacillus plantarum* HAN99 polysaccharides.

The FT-IR spectrum of the bacterial polysaccharide showed characteristic peaks from 400 to 4000 cm^−1^ (Fig. 2), indicative of its structural components. Stretching vibrations of hydroxyl (-OH) groups were observed at 3385.8 cm^[−1 [28,37,38^. The presence of C-H bonds, likely from CH_2_ groups in the polysaccharide backbone, was evident at 2931.7 cm^[−1 [39^. A prominent peak appears at 1647.92 cm⁻¹, which may be assigned to the asymmetric stretching vibration of carboxylate groups (COO⁻), suggesting the presence of uronic acid^40^. Additional bands at 1401.56 cm⁻¹ and 1232.16 cm⁻¹ could be attributed to symmetric COO⁻ stretching and C–O–H bending, further reinforcing the presence of uronic acid^40^. Studies support these findings about the possibility of the presence of uronic acid in polysaccharide produced by lactic acid bacteria^41,42^.

**Fig. 3:**
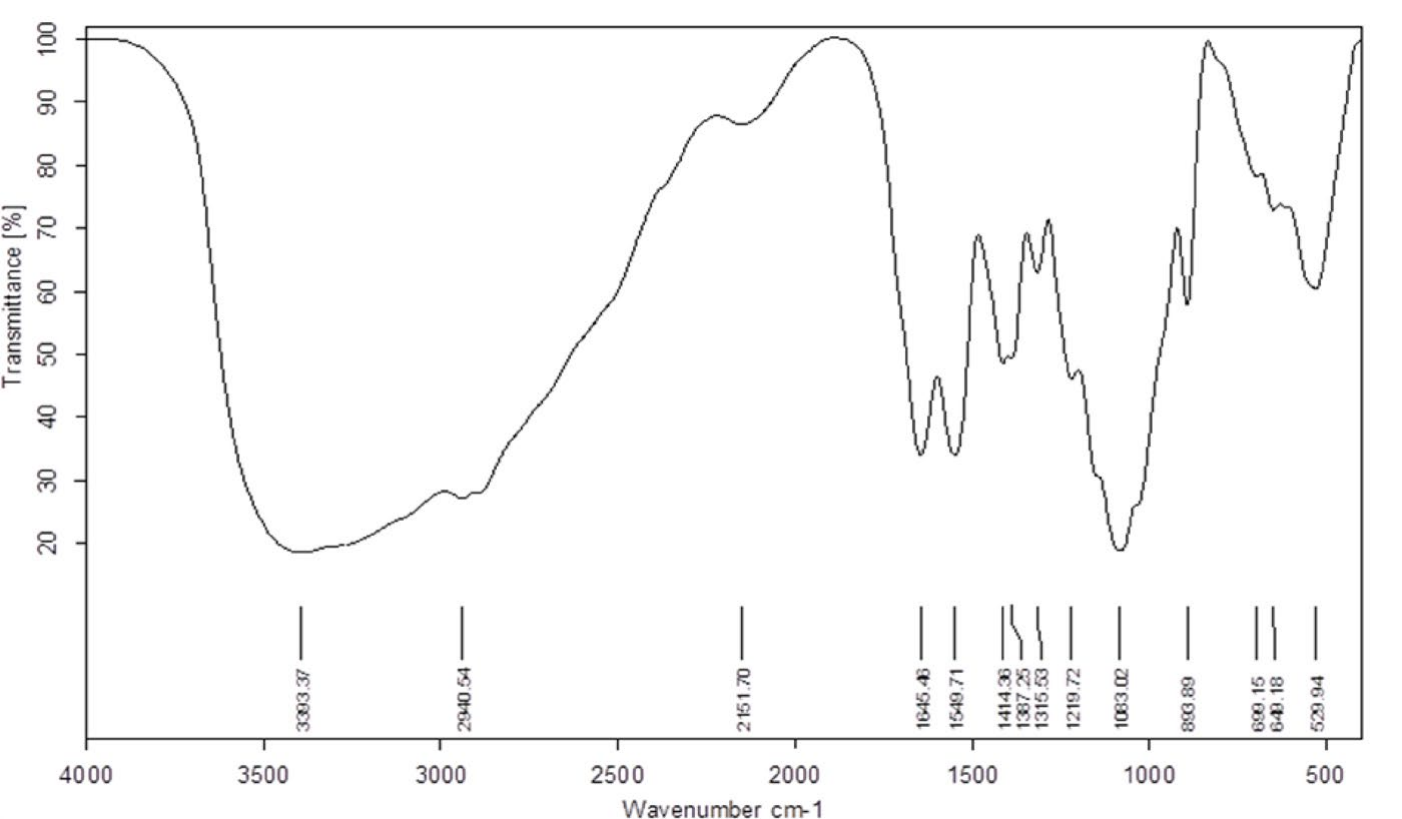
FT-IR analysis of *Lactiplantibacillus plantarum* HAN99 polysaccharide-based nanoparticles.

The FT-IR spectrum of chitosan-based nanoparticles was recorded to examine the structural differences between the polysaccharide and its nanoparticle form. A prominent peak at 3393.37 cm⁻¹ (Fig. 3) was detected, which corresponds to the stretching vibration of hydroxyl (-OH) groups. This peak is a characteristic feature common to both the polysaccharides and chitosan^43^. Notably, peaks corresponding to the stretching vibration of C-H bonds, primarily from CH_2_ and CH_3_ groups, were evident at 2940.54 cm^[−1 [44^. Furthermore, peaks associated with amino (N-H) groups (1549.71 cm^−1^)^43^ and stretching vibrations of C-H bonds in CH_3_ groups (1387.25 cm^−1^)^45^ were characteristic of chitosan. Similarly, the peak at 1645.46 cm^−1^, corresponding to the stretching vibration of carbonyl (C = O) groups in amides, was indicative of chitosan’s presence, specifically due to acetyl groups^45,46^. Notably, these characteristic functional groups were present exclusively in the FT-IR analysis of the nanoparticles, reflecting the incorporation of chitosan into the nanoparticle structure. In contrast, the FT-IR analysis of bacterial polysaccharides did not exhibit these distinctive chitosan-related peaks, underscoring the specificity of the spectral signature of each sample and highlighting the differences between the two analyses. A direct visual comparison of the two FT-IR spectra is presented in the overlay image (Supplementary Figure. 3), which clearly illustrates the morphological and spectral changes before and after nanoparticle synthesis.

### **Zeta potential measurement of*Lactiplantibacillus plantarum*HAN99 polysaccharide-based nanoparticles**

In this study, zeta potential measurements were conducted on the polysaccharide-based nanoparticles dispersed in water to assess their surface charge characteristics. The sample exhibited an overall positive zeta potential of + 37.9 mV, indicating a positively charged surface. This zeta potential is slightly higher than the 33.8 mV of polysaccharides extracted from *Pediococcus pentosaceus* by Jiang et al.,^47^. This result suggests a stable colloidal system because of the high zeta potential value (< + 30)^48^.

### Scanning electron microscope (SEM) study of the polysaccharide and synthesized chitosan-based nanoparticles

**Fig. 4:**
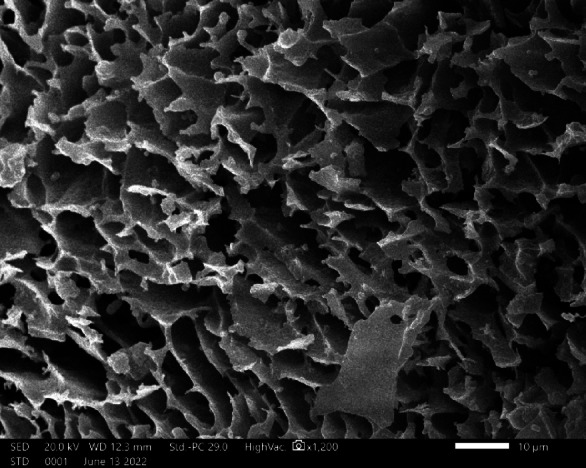
Scanning Electron Micrograph (SEM) of *Lactiplantibacillus plantarum* HAN99 polysaccharide at 1200X magnification.

**Fig. 5:**
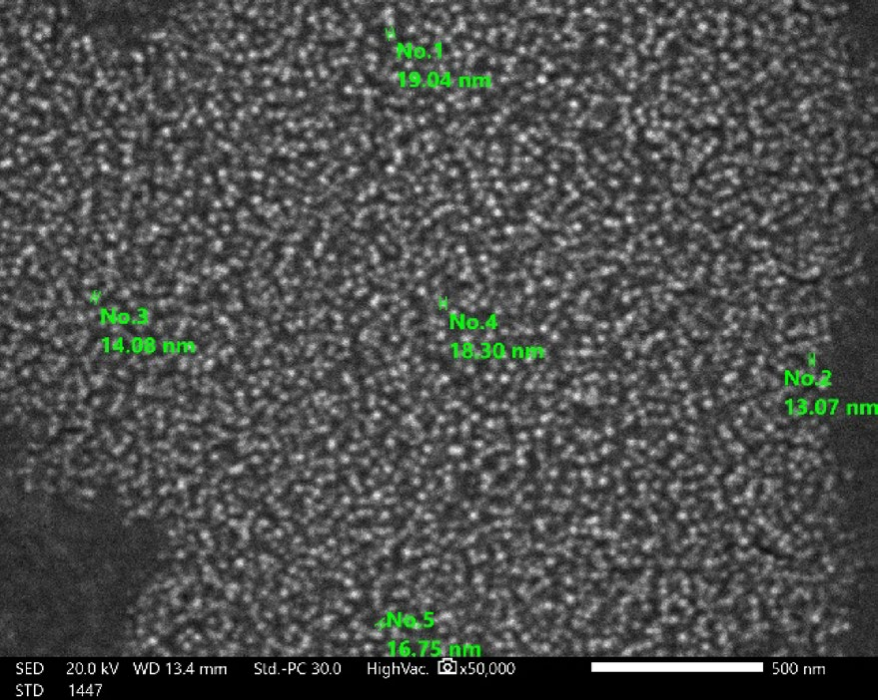
Scanning Electron Micrograph (SEM) of *Lactiplantibacillus plantarum* HAN99 polysaccharide-based nanoparticles at 50,000X magnification.

SEM analysis was performed twice on the polysaccharide to compare its natural structure with the newly synthesized chitosan-based nanoparticles. The original polysaccharide exhibited irregular shapes with coarse surfaces at magnifications of 1200 X (Fig. 4), aligning with the findings of Gawande et al.,^49^. Its flake-like appearance resembles the SEM micrographs of EPS derived from *Lactobacillus paracasei* M7^50^ and *Lactobaciullus fermentum* CFR 2195^51^. The rough surface and irregular shape of the polysaccharide are advantageous for forming network structures and branches^52^. Additionally, this characteristic improves attachment capabilities compared to a smoother surface^53^ and promotes great cell adhesion^54^. In contrast, the examination of the polysaccharide-based nanoparticles at a higher magnification of 50,000 X revealed uniform, circular particles with sizes ranging from 13 to 20 nm (Fig. 5). This observation is consistent with the results reported by Ilgu et al.,^55^ who documented uniform and well-dispersed chitosan nanoparticles. Representative particle size measurements of 20 nanoparticles, calculated from the SEM image using ImageJ, are provided in Supplementary Table 1.

### Effect of polysaccharide chitosan-based nanoparticles on the growth of *Mentha* leaves

To investigate the impact of polysaccharide-based nanoparticles from *Lactiplantibacillus plantarum* HAN99 on the Mentha (mint) leaf growth. Ten pots of mint plants were used including four sets of three replicas. One of the experimental sets was for control purposes, which was irrigated with distilled water. The other experimental sets were exposed to polysaccharide chitosan-based nanoparticles at concentrations of 0.1, 0.2, and 0.3 mg/ml for 72–144 h. The growth progression of the mint leaves was observed under these different conditions. This experiment is in agreement with the methodology of Mojeremane et al.,^24^ Merino et al.,^56^ and Tariq et al.,^57^.

The results revealed significant variations among the experimental groups. The group treated with 0.1 mg/ml polysaccharide had a mean initial leaf count of 40 leaves, which increased to 63 leaves after 72 h and decreased to 51 leaves after 144 h, resulting in 19% loss rate. Similarly, the group treated with 0.2 mg/ml polysaccharide had mean initial leaf counts of 40 leaves, which increased to 66 leaves after 72 h and declined to 52 leaves after 144 h, representing 21% loss. In contrast, the group treated with 0.3 mg/ml polysaccharide showed significant development, beginning with a mean of 65 leaves and increasing to 125 leaves after 72 h before decreasing to 101 leaves after 144 h, with a loss percentage of 19%. The detailed percentage of leaf loss across all treatment groups is provided in Supplementary Table 2.

This data highlights the varying degrees of growth caused by different concentrations of polysaccharide-based nanoparticles, with lower leaf loss percentages observed compared to the control group. The improved growth in plants treated with polysaccharide chitosan-based nanoparticles is likely due to their ability to retain water, enhance nutrient absorption, and boost plant defense mechanisms. Polysaccharides, especially in hydrogels, can form a protective layer around roots, helping plants retain moisture and access nutrients more effectively^58^. Additionally, nanoparticles allow for a gradual release of nutrients, ensuring efficient absorption over time. This controlled release may explain the increase in leaf count and the lower leaf loss observed in treated plants compared to the control.

The results of this study support the hypothesis proposed by numerous researchers that hydrogels encapsulating polysaccharides can enhance plant growth^59,60^.

Although a phytotoxicity study was not conducted in this work, future investigations should assess the potential toxicity of the synthesized nanoparticles to ensure their safe and effective application in biological systems.

### DPPH radical-scavenging activity

The antioxidant activity of the isolated EPS was determined using DPPH free radical scavenging. The DPPH experiment revealed considerable antioxidant potential in all EPS samples. The DPPH radical-scavenging activity showed an increase after the treatment with the microbial polysaccharide. The water-treated mint leaves have 70.5% scavenging of DPPH free radicals, consistent with the findings of Al-Suhaibani and Al-Kuraieef (2013)^61^. In comparison, the inhibition percentages for the polysaccharide chitosan- based nanoparticles treated mint leaves were 75.6%, 77.8%, and 80.3% scavenging of DPPH free radicals of the concentrations 0.1 mg/ml, 0.2 mg/ml and 0.3 mg/ml, respectively (Table 3).

**Table 3:** The antioxidant activity (DPPH%) of the isolated EPS extract of mint leaves before and after treatments.

Sample	DPPH free radical scavenging (%)
**Control** (Mint leaves extract treated with Dist, water)	70.5
**Set 1-**Mint leaves extract treated with (0.1 mg/ml) polysaccharide chitosan- based nanoparticles	75.6
**Set 2**- Mint leaves extract treated with (0.2 mg/ml) polysaccharide chitosan- based nanoparticles	77.8
**Set 3**- Mint leaves extract treated with (0.3 mg/ml) polysaccharide chitosan- based nanoparticles	80.3

## Materials and methods

### Sample collection, processing, isolation and identification of lactic acid bacteria (LAB)

Water and sediment samples were collected along the seacoast of Alexandria, Egypt, and placed into sterile Falcon tubes. The samples were subjected to serial dilution several times using a factor of 10. 100 µl aliquots were taken from each dilution and spread onto de Man Rogosa and Sharpe (MRS) agar plates supplemented with 0.5% CaCO3. The plates were then incubated anaerobically at 30 °C for 48 h. After incubation, twelve Gram-positive isolates forming clear zones of acid formation with catalase-negative activity were selected, purified using streaking methods, and preserved on MRS agar slants at 4 °C for routine use or maintained in 30% glycerol at − 80 °C for long-term storage^62^.

The production of exopolysaccharides by the twelve isolates was initially screened based on the appearance of a mucoid or viscous phenotype on MRS agar, indicating potential EPS synthesis^63^. For quantitative analysis, strains were cultivated in MRS broth at 37 °C for 30 hrs^64^. Extraction of the polysaccharide was carried out according to the method mentioned below. The resulting EPS pellet was dissolved in distilled water, and its concentration was determined using the phenol-sulfuric acid method with glucose as the standard^70^ Results were recorded for triplicate sets of each strain, as shown in Supplementary Table 3.

Out of the twelve bacterial isolates, the prospective LAB was tentatively identified using the API 50 CHL test kit (BioMérieux, Lyon, France), as described by the manufacturer. Results were obtained after 24 and 48 hrs of incubation at 30 °C. Interpretation of the fermentation profiles was facilitated using the computer-aided database API-WEB™ V.5.0 software. Furthermore, the selected isolate was subjected to 16S rRNA gene sequencing following the method described by Ameen et al.,^65^. A similarity search was performed in the GenBank database using the BLAST algorithm online tool (http://blast.ncbi.nlm.nih.gov/Blast.cgi).

### Extraction and purification of polysaccharides from LAB isolates

The polysaccharides producing LAB isolates were grown in MRS broth at 30 °C on a rotary shaker at 120 rpm for 48 hrs. Bacterial cells were collected by centrifugation at 8000×g for 20 min at 4 °C, and 14% trichloroacetic acid (TCA) was added to the supernatant. Denatured protein was precipitated by centrifugation, and ice cold absolute ethanol was added to the supernatant in a 2: 1 ratio for 24 hrs. Polysaccharides were precipitated out by another round of centrifugation. The precipitate was dissolved in H2O, dialyzed using a tubular cellulose acetate membrane (1000 Da cut-off, Sigma-Aldrich, Germany), lyophilized, and stored at −20 °C for further experiments^66,67^.

### Application of Plackett- Burman experimental design (PBD) for the improvement of polysaccharide production by *Lactiplantibacillus plantarum*

The Plackett–Burman experiment design was performed to select the most significant factors required for maximum polysaccharide production by the selected isolate^68^. The study examined twelve different factors, including the rate of shaking inoculum size, culture volume, incubation time, inoculum age, peptone, yeast extract, glucose, tween, K_2_HPO_4_, sodium acetate 3H_2_O, and MgSO_4_.7H_2_O. Each factor was examined at two different levels; high level (+ 1) and low level (−1), as detailed in Supplementary Table 4.

Plackett–Burman design follows the first-order model equation:

Y = β_0_ + Σ β_1_ X_i_.

Where “Y” is the measured response, “β_0_” is the model intercept, “β_1_” is the linear coefficient, and “X_i_” is the level of independent variables.

### Scanning electron microscope (SEM) of the polysaccharide of *Lactiplantibacillus plantarum* HAN99

The microbial polysaccharide’s surface morphology and microstructure were examined using scanning electron microscopy (SEM)^69^ (JEOL JSM-5400, Tokyo, Japan). The study was conducted again following the formation of polysaccharide-based nanoparticles to determine their size and distribution.

### Determination of the total carbohydrate of isolated polysaccharide

The analysis of total carbohydrates in the polysaccharide was conducted according to the method of Nielsen^70^. Absorbance was recorded at 490 nm against distilled water as blank and the total carbohydrate content was estimated from a standard curve prepared using glucose.

### Preparation of polysaccharide Chitosan -based nanoparticles

Polysaccharide-based nanoparticles were developed through ionic gelation^71^ utilizing chitosan as a carrier. Firstly, chitosan was added to a flask containing 1% acetic acid and dissolved in distilled water. The mixture was stirred with a magnetic stirrer at room temperature until all the chitosan had dissolved. Subsequently, sodium tripolyphosphate was added with continuous stirring. The polysaccharide solution was prepared by dissolving it in distilled water and was added dropwise to the chitosan mixture. The mixture was stirred overnight and subsequently centrifuged, washed, and freeze-dried to obtain the chitosan nanoparticles.

### Detection of monosaccharide composition of EPS by High-Performance liquid chromatography (HPLC) analysis

High-Performance Liquid Chromatography (HPLC) was used to determine the polysaccharides monosaccharide composition. The polysaccharide sample was acid hydrolyzed with 2 M trifluoroacetic acid at 120 °C for two hours in order to break it down into its monosaccharides component before analysis. The hydrolysate was then neutralized and filtered through a 0.22 μm membrane. To improve detection, the monosaccharides underwent derivatization with 1-phenyl-3-methyl-5-pyrazolone, allowing for separation on a reversed-phase column. HPLC was performed on a C18 reversed-phase column. The mobile phase used was a mixture of water and acetonitrile (75:25). The flow rate was maintained at 1.0 mL/min^72^.

### FT-IR analysis of the resulting polysaccharide and their nanoparticles

FT-IR analyses of the polysaccharide and its nanoparticles were carried out to detect variations in the sample peaks intensity within the range of 500 and 4000 cm-1. The samples were blended with potassium bromide, pressed into thin pellets, and subsequently analyzed using an FT-IR spectrophotometer^72^.

### Zeta potential measurement of polysaccharide-based nanoparticles

Measurement of zeta potential of the polysaccharide-based nanoparticles was performed using clear disposable zeta cells and water as the dispersant^73,74^.

### Effect of polysaccharide-based nanoparticles on the growth of *Mentha* (Mint) plants

A set of ten pots of mint plants was used to investigate the effect of polysaccharide-based nanoparticles on the growth of mint leaves. The potted mint plants were purchased from a local plant nursery in Alexandria, Egypt. Triplicate sets of mint plants were irrigated daily with different concentrations of the polysaccharide-based nanoparticles solution (0.1, 0.2, and 0.3 mg/ml). Additionally, another control set of mint plants pots was irrigated daily only with distilled water. The growth of mint leaves was monitored by recording the mean number of leaves in each set at 72-hrs. intervals over 144 h.

### Antioxidant activity of polysaccharide

The assay of 2,2-diphenyl-1-picrylhydrazyl (DPPH) radical-scavenging activity of the mint (Mentha) leaves was determined as described by Bahramikia and Yazdanparast^75^ before and after treatment with the microbial polysaccharide. Extracted mint solution with varying concentrations was mixed with DPPH in ethanol. The mixture was shaken vigorously and left for 30 min. at room temperature. The absorbance was measured at 517 nm using a spectrophotometer, with a blank containing distilled water and DPPH solution. The inhibitory activity was determined using the formula:

$$\:\text{DPPH\:(\%)\:=\:}\left(\frac{{A}_{\text{blank}}-{A}_{\text{Sample}}}{{A}_{\text{blank}}}\right)\times\:100$$

Where; A_blank_ represents the absorbance value of the control and, A_Sample_ represents the absorbance value of the tested solution.

## Conclusion

Our study demonstrated that the polysaccharide produced by *Lactiplantibacillus plantarum* HAN99, which was isolated from sediments along the Mediterranean Seacoast in Alexandria, consisted mainly of glucose and galactose, as per the results of the HPLC analysis. This polysaccharide was subsequently used in the formulation of nanoparticles. Our findings indicated that the nanoparticles produced exhibited positive effects on the growth of mint leaves. The percentage of leaf loss in the experimental groups ranged from 19 to 21%, which was significantly lower than the 30% loss recorded in the control group. Furthermore, the lactic acid bacteria demonstrated the ability to mitigate oxidative stress by scavenging DPPH. Therefore, *Lactiplantibacillus plantarum* HAN99 presents considerable potential as a source of EPS-based nanoparticles with antioxidants properties, which can be used in agriculture fields used as plant growth regulators.

## Electronic supplementary material

Below is the link to the electronic supplementary material.

Supplementary Material 1

## Data Availability

The 16S rRNA gene sequence of Lactiplantibacillus plantarum HAN99 generated during this study has been deposited in the GenBank repository under accession number PP150039 and is publicly available at https://www.ncbi.nlm.nih.gov/nuccore/PP150039.
