# Supplementary material for: Exopolysaccharide produced from Lactiplantibacillus plantarum HAN99 and its nanoparticle formulations in agricultural applications
Source: Sci Rep. 2025 Jun 1;15:19188. doi: 10.1038/s41598-025-03913-9 (PMC12127468; doi:10.1038/s41598-025-03913-9)
Supplement: Supplementary file 1 — Supplementary Material 1 [file 41598_2025_3913_MOESM1_ESM.docx]

**Exopolysaccharide produced from Lactiplantibacillus plantarum HAN99 and its nanoparticle formulations in agricultural applications**

Hania M. El-Messiry^1^, Amira M. Hamdan^2^, Nevine B. Ghanem^1^, Mohamed Hagar^3^

^1^Botany and Microbiology Department, Faculty of Science, Alexandria University, Egypt

^2^Oceanography Department, Faculty of Science, Alexandria University, Egypt

^3^Chemistry Department, Faculty of Science, Alexandria University, Egypt


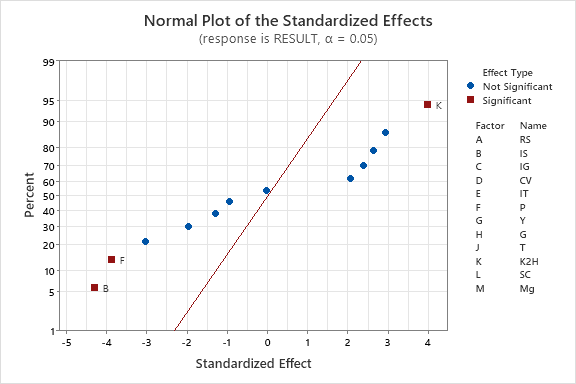


**Supplementary Figure 1:** Normal plot of the standardized effects showing the significance of variables inoculum size, peptone, K_2_HPO_4_ on the production of EPS by *Lactiplantibacillus plantarum* HAN99.

The normal probability plot of the standardized effects (P = 0.05) showed the significance of each factor and their interactions on the % of EPS production. This plot can be separated into two regions: the region with above 50% indicated as positive coefficients (K_2_HPO_4_) and the region below 50% indicated as negative coefficients (peptone and inoculum size).

| 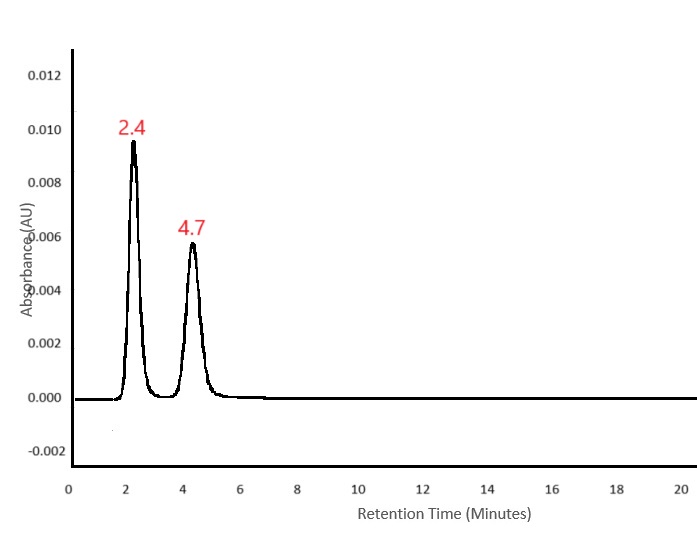  **Supplementary Figure 2.** HPLC chromatogram of the polysaccharide showing the retention times of the eluted sugars, glucose and galactose, respectively |
| --- |


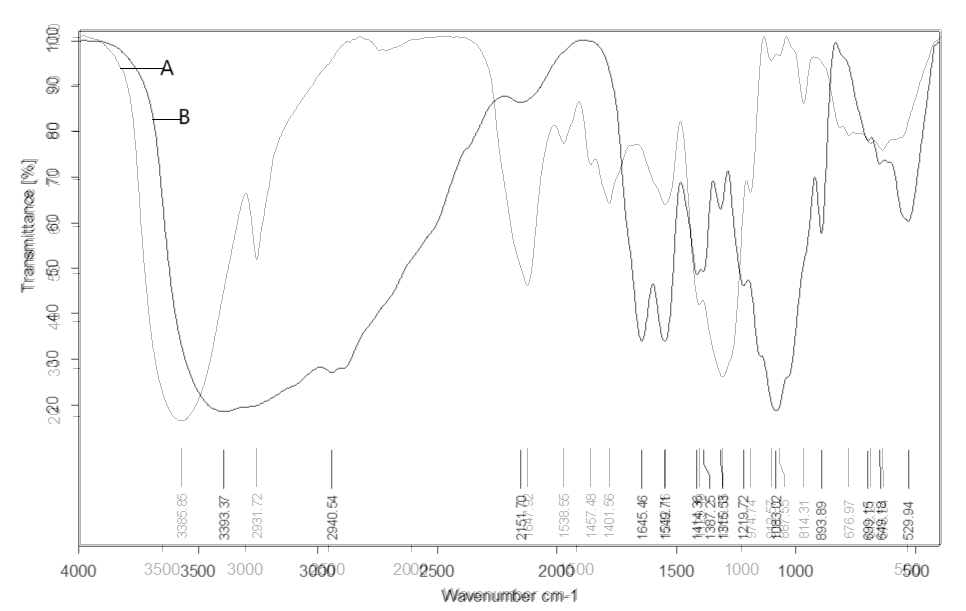


**Supplementary Figure 3:** Overlay of Figures 2 and 3 showing the morphological comparison of the polysaccharide (A) before nanoparticle synthesis and (B) after nanoparticle formation.

**Supplementary Table 1.** Representative particle size measurements of 20 nanoparticles analyzed from SEM image (Fig. 5) using ImageJ. Particle areas were measured in nm² and converted to estimated diameters assuming circular shape.

| **Particle** | **Area** |
| --- | --- |
| 1 | 11.5 |
| 2 | 20.909 |
| 3 | 10.455 |
| 4 | 11.5 |
| 5 | 23 |
| 6 | 15.682 |
| 7 | 12.545 |
| 8 | 15.682 |
| 9 | 11.5 |
| 10 | 11.5 |
| 11 | 13.591 |
| 12 | 10.455 |
| 13 | 48.091 |
| 14 | 14.636 |
| 15 | 17.773 |
| 16 | 19.864 |
| 17 | 13.591 |
| 18 | 25.091 |
| 19 | 12.545 |
| 20 | 11.5 |

**Supplementary Table 2.** The percentage of leaves loss of *Mentha* plant after treatment with the polysaccharide- chitosan -based nanoparticles of *Lactiplantibacillus plantarum* HAN99

| **Time (h.)** | **Mean Number of** *Mentha* (**Mint) Leaves** | | | |
| --- | --- | --- | --- | --- |
|  | **Control** | **Conc. of polysaccharide-chitosan based nanoparticles (mg/ml)** | | |
|  |  | **0.1** | **0.2** | **0.3** |
| **0** | **50** | **40** | **40** | **65** |
| **72** | **65** | **63** | **66** | **125** |
| **144** | **45** | **51** | **52** | **101** |
| **Leaf Loss (%)** | **30** | **19** | **21** | **19** |

**Supplementary Table 3.** Exopolysaccharide (EPS) yield of twelve *Lactiplantibacillus* strains after 30 hours of incubation in MRS broth at 37 °C. EPS concentrations were determined using the phenol-sulfuric acid method with glucose as the standard. Values are presented as mean ± standard deviation (n = 3).

| **Strain no.** | **EPS Yield (mg/ml)** |
| --- | --- |
| 1 | \| 18.7 ± 1.3 \| \| --- \|  \|  \| \| --- \| |
| 2 | \| 30.2 ± 1.8 \| \| --- \|  \|  \| \| --- \| |
| 3 | \| 35.9 ± 1.6 \| \| --- \|  \|  \| \| --- \| |
| 4 | 61.3 ± 2.5 |
| 5 | \| 48.2 ± 2.1 \| \| --- \|  \|  \| \| --- \| |
| 6 | \| 12.4 ± 1.1 \| \| --- \|  \|  \| \| --- \| |
| 7 | \| 22.9 ± 1.5 \| \| --- \|  \|  \| \| --- \| |
| 8 | \| 19.5 ± 1.0 \| \| --- \|  \|  \| \| --- \| |
| 9 | \| 43.6 ± 2.0 \| \| --- \|  \|  \| \| --- \| |
| 10 | \| 27.8 ± 1.4 \| \| --- \|  \|  \| \| --- \| |
| 11 | \| 38.4 ± 1.7 \| \| --- \|  \|  \| \| --- \| |
| 12 | \| 25.1 ± 2.2 \| \| --- \|  \|  \| \| --- \| |

**Supplementary Table 4.** Independent variables for maximum polysaccharides production by the selected LAB isolate using Plackett–Burman design

| **Independent Variables** |  |  | **Experimental level** | |
| --- | --- | --- | --- | --- |
|  | **Factor** | **Code** | **-1** | **+1** |
| **Incubation time (hrs)** | A | IT | 24 | 72 |
| **Rate of shaking (rpm)** | B | RS | 100 | 140 |
| **Culture volume (ml)** | C | CV | 100 | 500 |
| **Glucose (g/l)** | D | G | 15 | 25 |
| **Yeast extract (g/l)** | E | Y | 2 | 5 |
| **Peptone (g/l)** | F | P | 5 | 15 |
| **Tween (ml)** | G | T | 0.5 | 1.5 |
| **K_2_HPO_4._7H_2_O (g/l)** | H | K_2_H | 10 | 25 |
| **Sodium acetate 3H_2_O ( g/l)** | J | SC | 2 | 6 |
| **MgSO_4._7H_2_O (g/l)** | K | Mg | 1.5 | 3.5 |
| **Inoculum size (%)** | L | IS | 0.5 | 2 |
| **Inoculum age (hrs)** | M | IG | 12 | 36 |
